# A novel approach for the purification of aggregation prone proteins

**DOI:** 10.1371/journal.pone.0260143

**Published:** 2021-11-22

**Authors:** Austin Royster, Sheema Mir, Mohammad Ayoub Mir

**Affiliations:** Western University of Health Sciences, Pomona, California, United States of America; Instituto Butantan, BRAZIL

## Abstract

The protein aggregation is one of the major challenges of the biotechnological industry, especially in the areas of development and commercialization of successful protein-based drug products. The inherent high aggregation tendency of proteins during various manufacturing processes, storage, and administration has significant impact upon the product quality, safety and efficacy. We have developed an interesting protein purification approach that separates the functionally active protein from inactive aggregates using a detergent concentration gradient. The C-terminally His tagged nucleocapsid protein of Crimean Congo Hemorrhagic fever virus (CCHFV) has high aggregation tendency and rapidly precipitates upon purification by NiNTA chromatography. Using the new purification approach reported here, the freshly purified protein by NiNTA chromatography was further processed using a detergent gradient. In this new purification approach the active protein is retained in the low detergent concentration zone while the inactive aggregates are promptly removed by their rapid migration to the high detergent concentration zone. The method prevented further aggregation and retained the RNA binding activity in the native protein despite numerous freeze thaw cycles. This simple approach prevents protein aggregation by rapidly separating the preformed early aggregates and creating the appropriate microenvironment for correctly folded proteins to retain their biological activity. It will be of potential importance to the biotechnological industry and other fields of protein biochemistry that routinely face the challenges of protein aggregation.

## Introduction

A newly translated protein can adopt different types of conformational states within the cellular environment. The adopted conformation is determined not only by the intrinsic amino acid sequence, but also by potential interactions with other proteins in the cellular environment [1]. For example, the monomeric protein constituents of an oligomeric protein complex, and molecular chaperones that guide the co-translational folding into the native state, play critical roles in the adoption of unique biologically active and stable protein conformation [2, 3]. Failure of these guiding interactions and proteotoxic stresses such as heat shock can cause proteins to expose aggregation-prone hydrophobic regions that trigger the protein aggregation [4]. In addition, proteins are temporarily at increased risk of aggregation during transport over membranes or through pores [4]. While the acute global protein unfolding stresses can cause a broad aggregation driven by hydrophobic interactions of multiple unfolded proteins, the highly structured aggregates found in amyloids are due to β sheet or β-hairpin associations between single protein species [5].

Unlike eukaryotes the reduced environment of bacterial cytosol, lack of eukaryotic chaperones and post-translational machineries limit the efficient protein folding capability of a bacterial system. Due to these limitations, the huge expression of recombinant proteins in *Escherichia coli* often results in aggregation in inclusion bodies [6–8]. The expression of the recombinant proteins at high translational rates exhausts the bacterial protein quality control system, resulting in the aggregation of partially folded and misfolded protein molecules to form inclusion bodies [9]. Formation of inclusion bodies poses a great challenge in the production and purification of recombinant proteins using *E*. *coli* as host. Purification of native-like proteins from inclusion bodies is extremely difficult. Despite the intensive processing, including the isolation of inclusion bodies from the cell, solubilization using denaturants, followed by refolding, the finally purified proteins tend to re-aggregate and show minimal activity. Protein aggregation in inclusion bodies can be reduced by decreasing the temperature of growing bacterial culture up to 16°C and reducing the inducer concentration. Although these efforts can be helpful for certain protein, but for most others the aggregation is triggered when bacterial cells are lysed during the purification process.

Crimean-Congo Hemorrhagic fever virus (CCHFV) is a tick-borne nairovirus in the order *Bunyavirales*. Its infection causes severe hemorrhagic fever with a mortality rate of 30% to 80% in more than thirty countries worldwide [10–13]. Infection in humans usually occurs by either tick bites or direct contact with contaminated blood or tissue samples from the infected hosts [14, 15]. The viral genome is composed of three negative sense RNA segments (S, M and L), which encode nucleocapsid protein (N protein), glycoprotein precursor (GPC), and RNA-dependent RNA polymerase (RdRp), respectively [16]. N protein in association with the viral RNA (vRNA) and complementary RNA (cRNA) forms three nucleocapsids that serve as templates for transcription and replication of the viral genome. The X-ray crystallographic structure at 2.3 Å resolution reported that CCHFV N protein is composed of stalk and head domains [17–19]. Recently, we reported that stalk domain of CCHFV N protein harbors an RNA binding site in the stalk domain that specifically binds to the 5’ untranslated regions and plays a role in the preferential translation of viral mRNA [20].

Here, we report the purification of Crimean Congo Hemorrhagic fever virus (CCHFV) nucleocapsid protein using a detergent gradient approach that separates the early formed inactive protein aggregates from the correctly folded active protein and prevents further aggregation in the native protein sample. The purified protein is resistant to aggregation despite numerous freeze thaw cycles and retains activity. The approach can be customized for the high throughput purification of recombinant proteins that are prone to aggregation. This native protein purification method produces significant amount of pure protein without denaturation and refolding steps, commonly used in most protein purification procedures. The approach will be of potential significance to biotechnological industry that faces challenges of protein aggregation at different stages of development and commercialization of protein-based drug products [21].

## Materials and methods

### Cells and other reagents

*Escherichia coli* Rosetta (DE3) cells were from Stratagene and *Escherichia coli DH5α* competent cells were from Fisher Scientific. The HisTrap HP 5 ml columns were from Sigma and GE Healthcare. All other reagents used for the growth of bacterial cultures and purification of both wild type CCHFV N protein and its stalk domain were from Sigma. The high precision Streptavidin biosensors (SAX) were from ForteBio.

### Bacterial transformation

The E. coli cells were transformed by the standard heat shock transformation procedure. Briefly, frozen E. coli cells were thawed on ice for 5 minutes, followed by the addition of 1 μl of plasmid DNA (10 ng) and further incubation on ice for 30 minutes. The cells were placed on water bath (42°C) for 30 seconds, followed by incubation on ice for 2 minutes. Cells were incubated with 350 μl of LB media at 37°C for 1 hour and 20 μl of the resulting culture were spread on LB agar plates containing appropriate antibiotic. The plates were incubated at 37°C overnight and the bacterial colonies harboring the plasmid of interest were used for the downstream experiments.

### Purification of CCHFV N protein and stalk domain

The protein purification was carried out as previously reported [20, 22, 23]. Briefly, *Escherichia coli* Rosetta (DE3) cells (Stratagene), transformed with plasmids expressing either wild type CCHFV N protein or stalk domain were grown in 250 ml cultures at 37°C until the OD at 600 nm reached 0.5. The protein expression was induced by the addition of 0.5 mM Isopropyl β-D-1-thiogalactopyranoside (IPTG) to the bacterial culture, followed by further incubation at 18°C for additional 20 hours. Cell pallets were re-suspended in 45 ml of lysis buffer (50 mM Tris-HCl, pH 7.4, 150 mM NaCl, 2 mM dithiothreitol [DTT], 0.5% Triton-X100, 5mM CHAPS, 0.1 mM phenyl methyl sulfonyl fluoride [PMSF]), followed by sonication on ice and clearance of the lysate by centrifugation at 4500xg for 20 minutes. The protein purification was carried out on AKTA pure protein purification system (GE Healthcare). The cleared cell lysates were loaded onto HisTrap NiNTA column having 5ml bed volume (Sigma), pre-equilibrated with the lysis buffer. The column was then washed with 50 ml of lysis buffer, followed by additional washing with 100 ml of wash buffer (50mM Tris, 500 mM NaCl, 0.05% Triton X-100 and 20 mM Imidazole). The bound protein was finally eluted by Imidazole gradient from 0 mM to 250 mM in the elution buffer (50mM Tris, 500 mM NaCl, 0.05% Triton X-100).

### Protein purification by the detergent gradient

The bacterial cultures were grown similarly as mentioned above. The bacterial pellets from 250 ml of the culture were suspended in 45 ml of lysis buffer (50 mM Tris-HCl, pH 7.4, 150 mM NaCl, 2 mM dithiothreitol [DTT], 1% Triton-X100, 5mM CHAPS, 0.1 mM phenyl methyl sulfonyl fluoride [PMSF]), followed by sonication and centrifugation as mentioned above. The clear lysates were loaded on HisTrap column, followed by washing with 100 ml wash buffer (50 mM Tris-HCl, pH 7.4, 500 mM NaCl, 1% Triton-X100, 20 mM Imidazole). The bound protein was finally eluted by Imidazole gradient from 0 mM to 250 mM in the elution buffer (50 mM Tris-HCl, pH 7.4, 500 mM NaCl, 1% Triton-X100). Each fraction was immediately diluted with equal volume of dilution buffer (50 mM Tris-HCl, pH 7.4, 1% Triton-X100, 0.4% Tween-20, 2 mM L-Arginine, 2 mM L-Glutamine). After dilution none of the fractions showed any turbidity and signs of visible aggregation. Each diluted fraction was frozen at -80°C overnight to allow the formation of detergent gradient. The active protein retained at the top of the gradient in the low detergent concentration zone was carefully removed and tested for RNA binding, as discussed in the results section.

### T7 Transcription for the synthesis of 5’UTR

The sequence encoding the short viral S-segment mRNA derived 5’ untranslated region (5’UTR) was PCR amplified from a plasmid DNA harboring the viral mRNA 5’ UTR, using a forward primer containing a flanking T7 promoter (5’TAATACGACTCACTATAGTCTCAAAGAAACACGUG3’) and a reverse primer (5’GTGTCACAAGAGAACTCA3’). The PCR product was gel purified and used as template in *in vitro* T7 transcription reaction. RNA synthesis was carried out using the T7 RiboMax kit (Promega), following the manufacturer’s instructions. The RNA was biotinylated as previously reported [22–24].

### Plasmids

The plasmid expressing the wild type CCHFV N protein from strain 10200 was a gift from Stuart T Nichol (CDC, Atlanta, Georgia). The N protein ORF was PCR amplified and cloned between NdeI and XhoI restriction sites in pET30a backbone to generate the plasmid pET-CCHFNP, as previously reported [22]. The region encoding the stalk domain of CCHFV N protein (amino acids 180–300) was PCR amplified. The PCR product was gel purified and cloned in pET30a vector between NdeI and XhoI restriction sites to generate pETstalk plasmid, as previously reported [20].

### Biolayer Interferometry (BLI)

Biolayer interferometry was used to monitor the binding affinities of wild type CCHFV N protein and its stalk domain with the 5’ untranslated regions (5’ UTR) of CCHFV S-segment derived mRNA, using the BLItz system (ForteBio Inc.), as previously reported [22–24]. Briefly, the biotin- 5’ UTR was synthesized and loaded onto high precision streptavidin biosensors (catalog # 18–5019, Forte Bio Inc.), as previously reported [20]. All reactions were carried out at room temperature in RNA binding buffer (20 mM Tris-HCl, pH 7.4, 80 mM NaCl, 20mM KCl, and 1mM DTT). After mounting the RNA, the biosensors were equilibrated in RNA binding buffer and then dipped in the purified protein solutions of either wild type N protein or stalk domain for the measurement of association kinetics. The reaction cycles were as follows: initial base line for 30 seconds, loading of biotinylated RNA on streptavdin biosensors for 120 seconds, base line for 30 seconds, association of protein with the RNA for 300s, followed by dissociation phase of 500 seconds. The kinetic parameters K_ass_ (association rate constant), K_dis_ (dissociation rate constant) and the binding affinities (Kd = K_dis_/K_ass_) were calculated with the help of inbuilt data analysis software (BLItZ Pro), as previously reported [22–24].

## Results

### Aggregation tendency of CCHFV N protein

**Fig 1:**
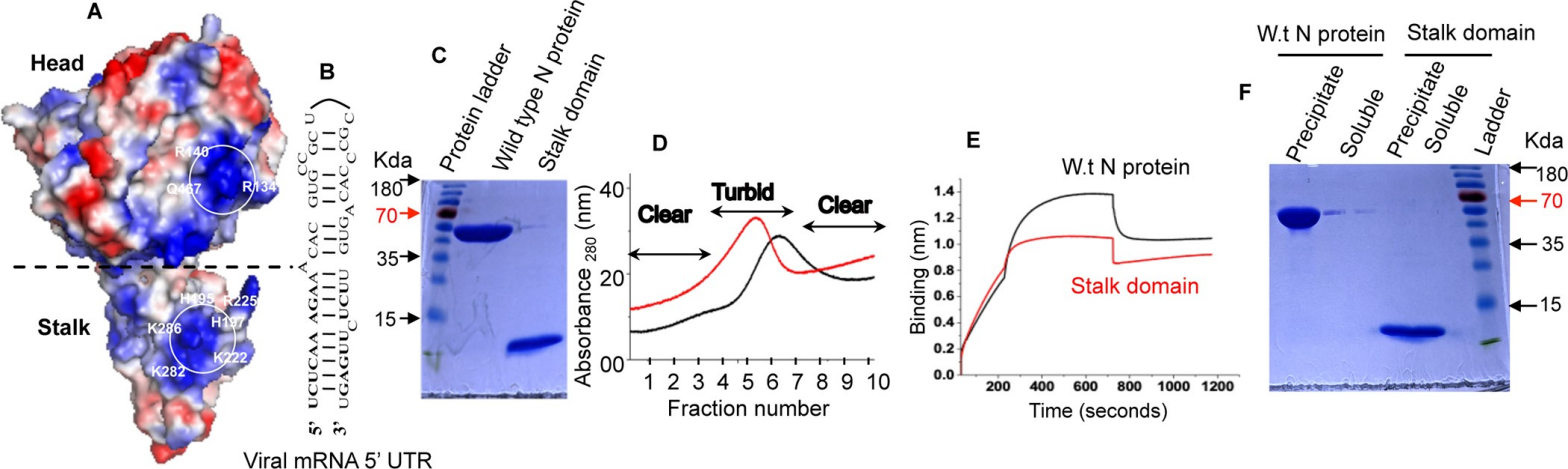
(**A**) A modeled 3D structure of CCHFV N protein showing head and stalk domain. The positive and negative charged surfaces are colored in blue and red, respectively. This model was previously reported in [20] where the amino acid composition of RNA binding sites in the stalk and head domains is discussed. (**B**) A panhandle like secondary structure formed by the partially complementary nucleotides of the 5’ UTR of the CCHFV S-segment derived mRNA. (**C**) Elution profile of wild type N protein (red) and stalk domain (black) from the HisTrap NiNTA column using the AKTA pure protein purification system (GE Healthcare). The clear and turbid fractions are shown by arrows. (**D**) The clear fractions 2 and 9 from panel C were combined and tested for RNA binding activity. Shown are the Biolayer interferometry (BLI) sensograms of wild type N protein (black) and stalk domain (red), demonstrating the association and dissociation kinetics for protein-RNA interaction. (**F**) The precipitated wild type N protein and stalk domain were briefly centrifuged at room temperature. The protein in the precipitated pellet and the supernatant (soluble) were examined by SDS-PAGE.

The previously reported X-ray crystal structure showed the presence of head and stalk domains in the structure of CCHFV N protein (Fig 1A). Using a native purification protocol, we expressed both wild type N protein and stalk domain in *Escherichia coli* and purified them by NiNTA chromatography using the C-terminal His tag, as previously reported [20, 22, 23] and discussed in Materials and methods. The eluted proteins (wild type and stalk domain) were pure and matched correctly with their respective molecular weights on SDS-PAGE (Fig 1C).

The eluted protein fractions containing highest concentration of either wild type protein or stalk domain became turbid immediately upon elution, showing signs of aggregation (Fig 1D). However, the fractions containing lower concentrations of the protein were clear and showed no visible turbidity. The eluted fractions were assayed for RNA binding using Biolayer Interferometry, before their storage at -80°C. Due to sequence complementarity the 5’ UTR internally folds into a partially base paired panhandle like secondary structure (Fig 1B). As shown in Fig 1E both wild type protein and stalk domain in clear fractions bound to the viral mRNA 5’ UTR with a Kd ~ 54 ± 10 nM, consistent with previously published reports [20, 22, 23]. In comparison, the turbid fractions of both wild type protein and stalk domain showed no binding with viral mRNA 5’ UTR. After overnight storage at -80°C the clear fractions were thawed at room temperature for one hour. Interestingly, the protein rapidly aggregated into white visible precipitate after thawing. The precipitate was pelleted down, and the supernatant was tested for any soluble protein by SDS-PAGE. Fig 1F clearly demonstrates that all the protein had aggregated leaving no soluble protein in the solution. Similar aggregation problem was observed in our previous studies where the interaction of purified stalk domain and wild type protein with viral mRNA 5’ UTR was studied in detail [20, 22, 23]. Due to the aggregation problem, the freshly prepared protein was preferably used for RNA binding, and the unused soluble protein was ten fold diluted before storage at -80°C to avoid aggregation during the freeze thaw cycle. However, the highly diluted protein needed to be concentrated before using it second time in RNA binding experiments. Nonetheless, none of the protein was useful after the 1^st^ freeze thaw cycle due to aggregation problem, making this purification approach less useful especially for processes requiring the storage of large quantities of protein at -80°C.

### Purification by detergent gradient and RNA binding activity of diluted protein fractions

**Fig 2:**
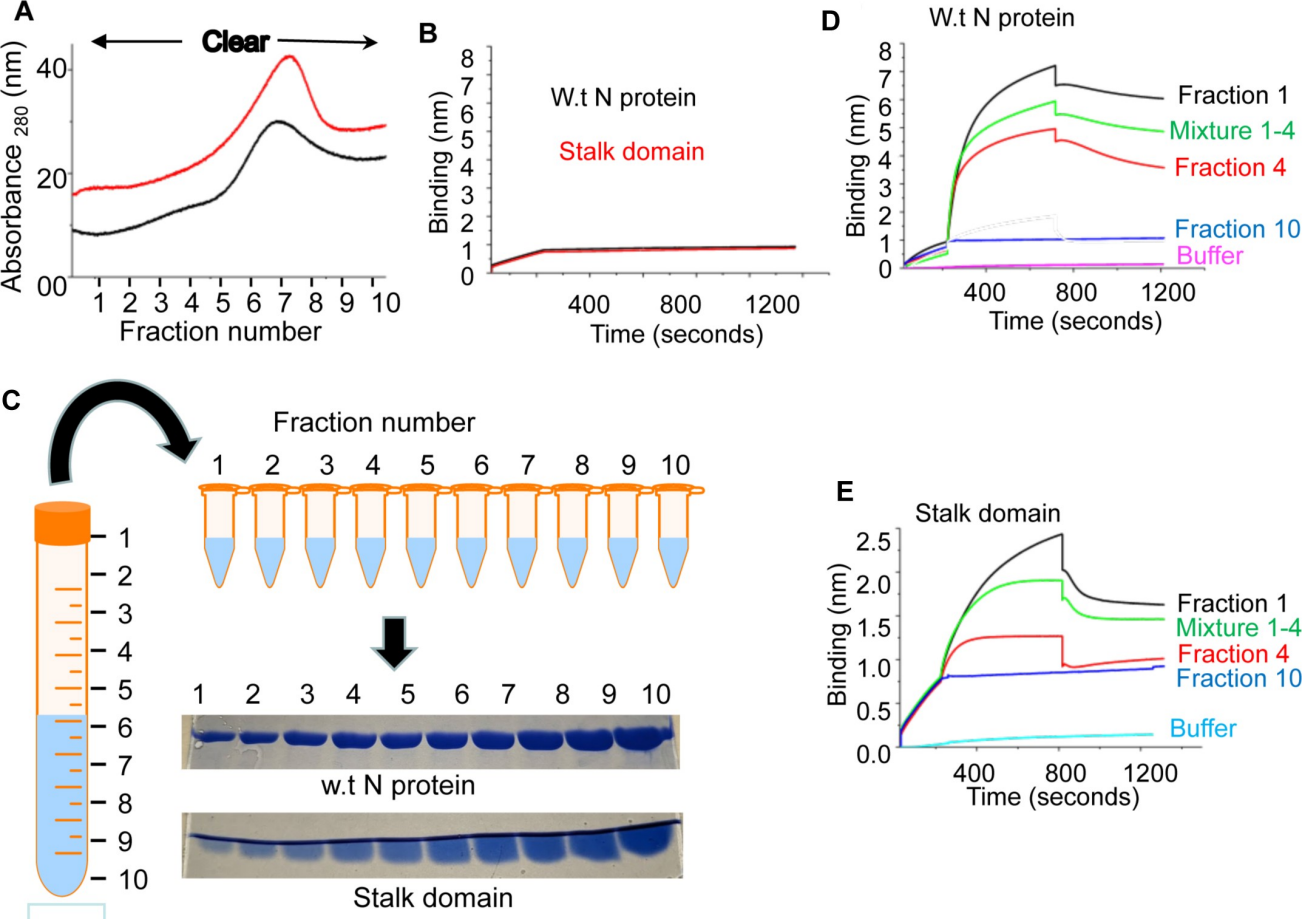
(**A**) Elution profile of wild type N protein (red) and stalk domain (black) using the new purification method on AKTA pure protein purification system. (**B**) The fractions from panel A were 50% diluted with dilution buffer and assayed for RNA binding activity. Shown are the BLI sensograms of wild type N protein (red) and stalk domain (black) for binding to the RNA. (**C**) The diluted fraction of 10 ml volume is thawed at room temperature after overnight freezing at– 80°C. The number line shows the 1 ml markings on the tube. The 1 ml fractions from 10 ml tube are poured into 1 ml Eppendorf tubes without disturbing the concentration gradient, as shown. SDS-PAGE analysis of the 1 ml fractions is shown. (D & E) The representative BLI sensograms for RNA-protein interaction of some of the fractions in panel C are shown. Wild type N protein (D) and stalk domain (E) from respective fractions 1,4,10 and mixture 1–4 at a concentration of ~ 250 nM and ~ 150 nM were used to generate the shown BLI sensograms, demonstrating the association and dissociation kinetics for N protein-RNA interaction. The fraction 1 in panel D is also shown in Fig 4C as P1 for comparison.

Both N protein and stalk domain were repurified by increasing the Triton-X100 concentration in lysis buffer, wash buffer and elution buffer (see protein purification by the detergent gradient in Materials and methods). The elution profile of both wild type N protein and stalk domain was similar to the previous procedure (compare Fig 1D with Fig 2A), except that wild type N protein eluted at slightly higher imidazole concentration during this new procedure. Each fraction was immediately diluted with equal volume of dilution buffer (see Materials and methods), following which none of the fractions showed any turbidity and signs of visible aggregation before and after freezing at -80°C. However, without the addition of dilution buffer, the fractions were clear before freezing but rapidly precipitated after a single freeze thaw cycle.

The diluted protein fractions were tested for RNA binding activity before freezing at -80°C using the Biolayer Interferometry, as mentioned in Materials and methods. Surprisingly, none of the fractions showed any binding to the viral mRNA 5’ UTR (Fig 2B). The diluted fractions having a total volume of 10 ml each were then frozen overnight at -80°C. Next morning the fractions were thawed at room temperature for 1 hour. After thawing, each fraction showed a visible density gradient from top to bottom which became more apparent upon slightly shaking the solution. The solution from each 10 ml tube was carefully poured out in 1ml fractions from top to bottom without disturbing the gradient (see Fig 2C). SDS-PAGE analysis showed that concentration of both wild type N protein and stalk domain gradually increased towards the bottom of the gradient (Fig 2C). Each fraction was assayed for RNA binding activity using Biolayer interferometry. As show in the representative sensograms (Fig 2D & 2E), the wild type N protein and stalk domain in top fractions was highly active. Although the dissociation constant for RNA-protein interaction for different fraction was similar (Table 1), the amount of active protein gradually decreased from top to the bottom of the gradient, evident from decreased binding (compare fraction 1 with fraction 4 in Fig 2C & 2D).

**Table 1:** Binding parameters for the association of wild type N protein and its stalk domain with the 5’ UTR of S-segment derived mRNA.

Protein Name	Kd (*nM*)	K_ass_ (*m*^*-1*^ *s*^*-1*^)	K_dis_ (*s*^*-1*^)
Stalk Fraction 1	54.24 ± 10	1.08x10^5^	5.87x10^-3^
Stalk Fraction 4	52.48 ± 16	3.11x10^5^	1.82x10^-2^
Stalk Mixture 1–4	36.67 ± 3	1.40x10^5^	5.10x10^-3^
Wild Type Fraction 1	28.71 ± 1	5.84x10^4^	1.57x10^-3^
Wild Type Fraction 4	45.91 ± 2	4.95x10^4^	2.28x10^-3^
Wild Type Mixture 1–4	37.26 ± 1	1.16x10^5^	4.93x10^-3^

Note: Kd = K_ass_ / K_dis_

We next mixed the 20 ul of protein sample from each fraction, and the resulting mixture was assayed for RNA binding activity. Again, the mixture generated from fractions 1–10 showed no RNA binding activity. Similar results were obtained when the gradient was disturbed by briefly vortexing the solution in the 10 ml tube before fractionation.

**Fig 3:**
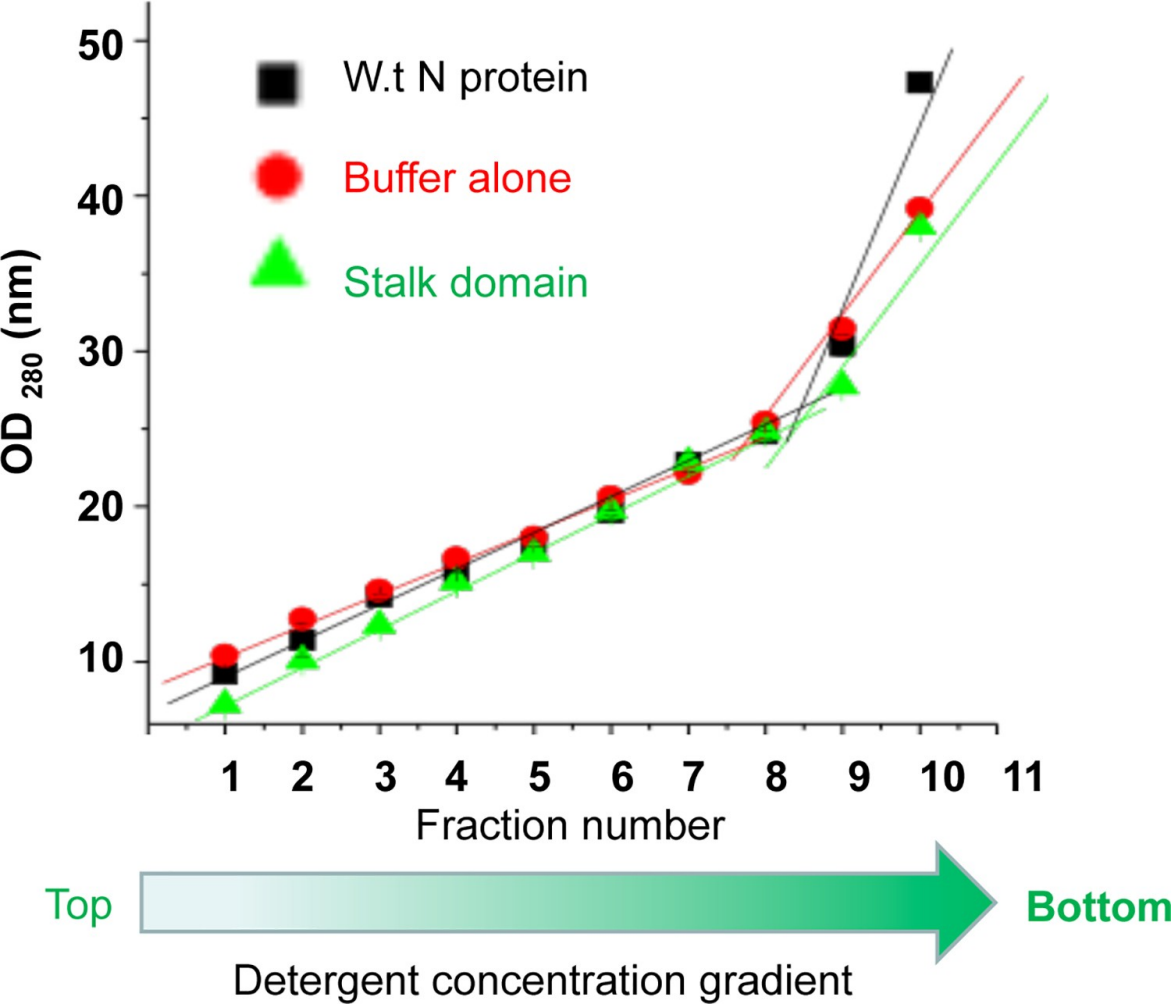
The absorbance at 280 nm of the fractions 1–10 from buffer alone (red), wild type N protein (black) and stalk domain (green) were recorded and plotted verses fraction number. The data points (fractions 1–7) and (fractions 8–10) were separately fit to straight lines, showing two different slops.

To ensure the formation of detergent gradient, a control purification experiment was carried out in which lysis buffer devoid of bacterial pellet, was subjected to all purification steps and the final elution’s were 50% diluted with dilution buffer as mentioned above. The eluted samples were frozen overnight at -80°C and thawed next morning. The thawed solution from 10 ml tubes was carefully poured out in 1 ml fractions from top to bottom without disturbing the gradient. Since triton X-100 absorbs at 280 nm, the OD measurements at 280 nm for each fraction was recorded to gauge insight about the triton X-100 concentration in different fractions. As shown in Fig 3, the triton X-100 concentration gradually increased from top to bottom, demonstrating the formation of a concentration gradient.

The fractions from wild type N protein and stalk domain showed a similar increase in OD measurements from top to bottom of the gradient, although the presence of protein contributed to the absorption signal at 280 nm. This experiment suggests that active protein remains in the low detergent concentration zone towards the top of the gradient, and the inactive protein moves towards the high detergent concentration zone towards the bottom of the gradient. Thus, detergent gradient helped to separate the active and inactive protein from the original sample.

### Impact of the detergent upon N protein activity

**Fig 4:**
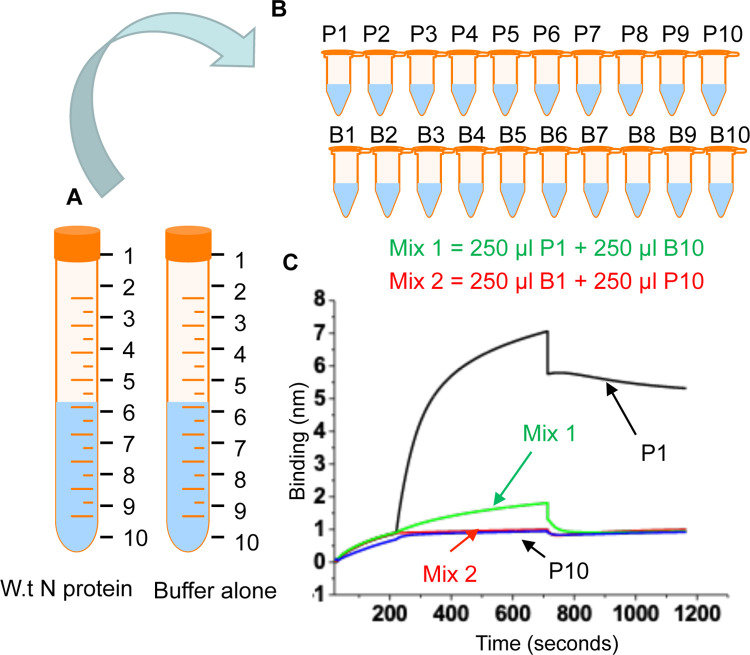
(**A**) Wild type N protein eluted from the column was diluted 50% by the dilution buffer. A diluted fraction (10 ml) was frozen overnight at -80°C and thawed next morning. The lysis buffer devoid of N protein was subjected to similar purification steps and a diluted fraction (10 ml) was similarly frozen overnight and thawed next morning, shown as buffer alone. (**B**) The defrosted N protein solution and buffer alone from panel A were poured into Eppendorf tubes in 1 ml fractions (P1-P10) and (B1-B10), respectively, from top to bottom of the concentration gradient. (**C**) BLI sonograms for the binding of N protein from fractions P1 (250 nM), P10 (250 nM), Mix 1 (125 nM) and Mix 2 (125 nM) with RNA are shown.

We next wanted to confirm that loss of activity in wild type N protein at the bottom of the gradient was not due to higher concentration of the detergent. The wild type N protein sample from fraction 10 corresponding to the bottom of the gradient was diluted using the fraction 1 corresponding to the top of the gradient devoid of N protein, and the resulting mixture, shown as Mix 2 in Fig 4, was tested for RNA binding. It is evident from Fig 4 that dilution had no impact upon RNA binding activity (compare Mix 2 with P10 in Fig 4), suggesting that N protein in the high detergent concentration zone at the bottom of the gradient is likely aggregated and thus inactive.

Similarly, the active protein in fraction 1 corresponding to low detergent concentration at the top of the gradient was diluted by the fraction 10 of the detergent gradient devoid of N protein, and the resulting mixture, shown as Mix 1 in Fig 4, was tested for RNA binding. As shown in Fig 4 that dilution by fraction 10 having high concentration of the detergent adversely affected the RNA binding activity of the Mix 1 (compare Mix 1 with P1 in Fig 4). It must be noted that both Mix1 and Mix2 contain wild type N protein from fractions 1 and 10, respectively, and the total detergent concentration in both the mixtures should be the same. Since Mix 1 retained the residual RNA binding activity compared to Mix 2, it further suggests that N protein at the bottom of the gradient is inactive likely due to aggregation. Nonetheless, the separation of protein in high and low concentration zones of the detergent prevented further aggregation and precipitation of the protein. The separation rendered the protein highly active and resistant to aggregation in the lower detergent concentration zone towards the top of the gradient.

### The purified protein by detergent gradient is stable during freeze thaw cycles

**Fig 5:**
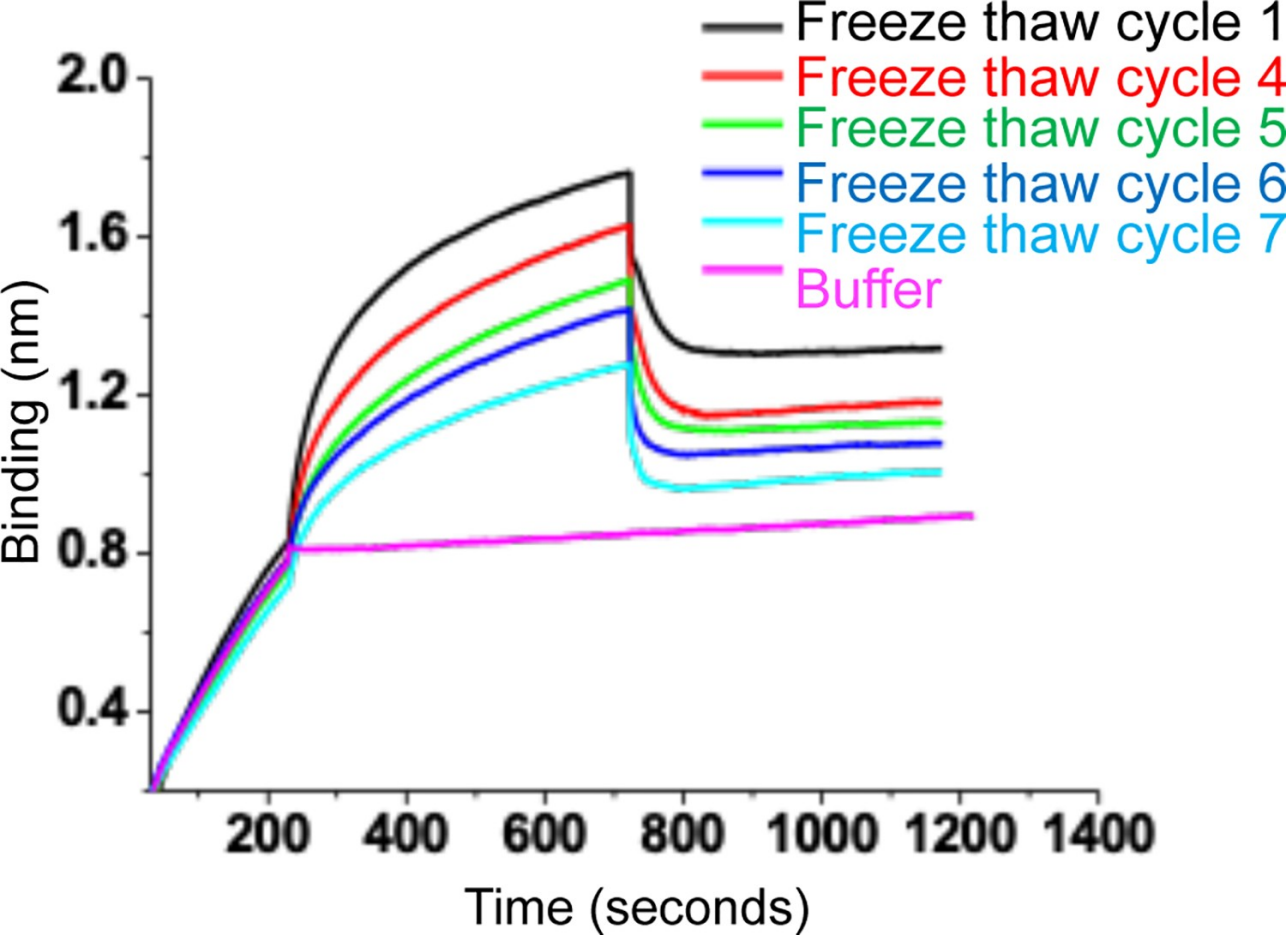
A mixture of wild type N protein generated by mixing equal volumes of fraction 1–4 (Fig 2) at a final concentration of 125 nM was tested for RNA binding after different freeze thaw cycles. The BLI sensograms in different colors demonstrating association and dissociation kinetics of N protein with the RNA after different freeze thaw cycles are shown.

The wild type N protein at the top of the gradient (mixture of fractions 1–4) was frozen overnight at -80°C and thawed in the morning at room temperature for 1 hour, followed by the examination of RNA binding activity. The Freeze thaw cycles were repeated multiple times. As shown in Fig 5, the freeze thaw cycles had moderate impact upon the activity, the protein solution was transparent without any visible sign of aggregation and showed RNA binding activity even after seven freeze thaw cycles. Although the dissociation constant for N protein-RNA interaction (Kd ~ 67 ± 5 nM) was not affected, the quantity of active protein slightly decreased at every freeze thaw cycle (Fig 5).

### Requirement of freeze thaw cycle for detergent gradient formation

**Fig 6:**
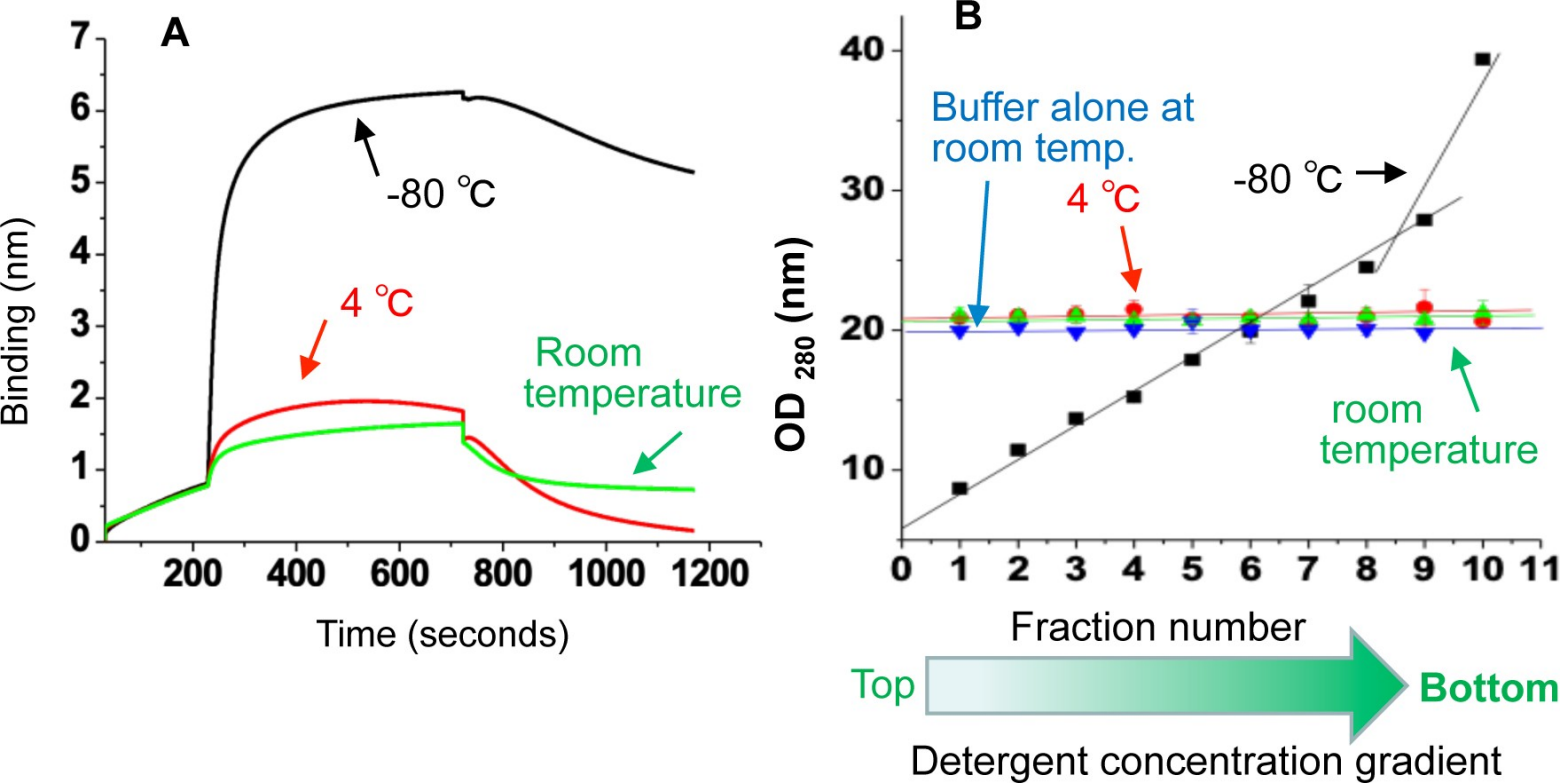
(**A**) The eluted fractions of the wild type N protein were 50% diluted and either frozen over night at– 80°C or left at room temperature or at 4 oC. Next morning that thawed solution were poured into 1 ml fractions from top of the gradient. Shown are the BLI sensograms of N protein from the fraction 1, demonstrating the association and dissociation kinetics with the RNA. (**B**). The OD measurements of each fraction at 280 nm was carried out as mentioned in Fig 3. The OD values corresponding to the fraction number were plotted and the data points were fit to a straight line.

Wild type N protein was purified as mentioned in Fig 2. The eluted fractions from the column were immediately 50% diluted with dilution buffer. The fractions were either frozen over night at -80°C or left at room temperature or at 4°C overnight. Next morning the fractions were poured into 1 ml fractions from top to bottom, as mentioned in Fig 2C. The fractions were assayed for RNA binding activity using BLI. It is evident from Fig 6A, that N protein in the top fraction from frozen sample contained highest amount of active N protein that showed strong RNA binding activity.

However, the top fractions from samples left at either room temperature or at 4°C showed RNA binding activity, but the amount active protein was dramatically reduced. To determine whether the detergent concentration gradient was formed in the samples, each fraction was tested for absorbance at 280 nm. Interestingly, the frozen protein sample formed the triton X-100 concentration gradient, evident form the positive slop of the straight line fit to the data points. However, the free buffer or N protein samples left at room temperature or at 4°C failed to form such concentration gradients. This clearly suggest that freeze thaw cycle is required to form the detergent concentration gradient in which active protein stays at the top of gradient towards the low detergent concentration zone and the inactive aggregated protein moves down to the high detergent concentration zone of the gradient towards the bottom.

## Discussion

*Escherichia coli* remains the dominant host for the production of recombinant proteins due to numerous advantages, such as, high yield protein production, ease of use, fast growth of the bacterial cultures, cost effectiveness, together with the well characterized genetics and a variety of available molecular tools. The recombinant proteins produced from E. coli have significantly advanced the general filed of biology and biopharmaceutical industry. For example, about 90% of the protein whose structures have been reported in the protein data bank and about 30% of the recombinant biopharmaceuticals licensed for use were produced in E. coli [25–28]. The major disadvantage of this bacterial system for the production of recombinant proteins is the poor solubility of the protein of interest, resulting in the accumulation and thus purification from the inclusion bodies by stringent non-native purification methods. About 75% of human proteins are expressed in bacteria but only 25% are produced in active soluble form [29, 30]. While solubility remains the major bottleneck in the field, the issue can be handled by fusing the recombinant protein with a protein tag such as glutathione S-transferase (GST), a 26 KDa protein that rapidly folds into highly stable and soluble protein upon translation. The tag can be enzymatically removed after the purification for downstream applications of the recombinant protein. The CCHV N protein was previously expressed in soluble form as a GST fusion protein, followed by the proteolytic removal of the GST tag after purification, and the use of N protein in X-ray crystallographic studies [17–19].

Interestingly, the N protein fused to a C-terminal His tag was highly expressed in bacteria but it rapidly aggregated after purification by the native purification procedure. The aggregation tendency was so high that fractions containing the high protein concentration became turbid immediately after purification and lacked the RNA binding activity. Although clear protein fractions containing low concentrations of the protein showed some RNA binding activity, but the protein was rapidly aggregated and lost activity by a single Freeze thaw cycle. Such high aggregation tendency could be due to protein misfolding, creating hydrophobic patches on the protein surface that triggered rapid aggregation and precipitation at higher concentrations. Increasing the triton X-100 concentration to 1% in all purification buffers prevented the formation of visible aggregates but the protein remained functionally inactive evident from the lack of RNA binding activity. This suggests that triton X-100 prevented further aggregation by blocking the hydrophobic patches but didn’t facilitate protein refolding into functional form. It is equally possible that presence of invisible aggregates of varying sizes prevented the binding of correctly folded functional protein, if any in the mixture, to the RNA. Fifty percent dilution of eluted protein fractions by the dilution buffer containing L-Arginine, L-Glutamine and higher detergent concentration effectively prevented the formation of visible protein aggregates by reducing the protein concentration and by blocking the hydrophobic patches on misfolded protein. In addition, L-Argine and L-Glutamine are known to promote protein folding [31]. Thus, it is likely that dilution buffer also helped in the correct folding of misfolded protein. However, the lack of activity in the diluted fractions was likely due to the interference of invisible aggregates in RNA binding. Interestingly, a single freeze thaw cycle of diluted fractions triggered the formation of a detergent concentration gradient in which highly active protein migrated to the low detergent concentration zone towards the top of the gradient, while the inactive protein migrated to the high detergent concentration zone towards the bottom of the gradient. This observation supports the idea that purified protein fractions contain a mixture of both active and inactive forms of the protein. The inactive misfolded form has high aggregation tendency and its presence in the mixture impacts the RNA binding activity of the active form. Nonetheless, the detergent gradient draged the inactive N protein towards the high detergent concentration zone, leaving the active protein towards the low detergent concentration that retained activity without further aggregation. Interestingly, the active protein in the low detergent concentration zone was fairly stable at multiple freeze thaw cycles.

The concentration of both Triton X-100 and Tween-20 in all eluted fractions (Fig 2C) is above the critical micelle concentration (CMC), the minimum detergent concentration at which micelle formation is initiated [32, 33]. A typical micelle in water is an aggregate of surfactant molecules (Triton X-100 and Tween-20 in this case), when their hydrophilic head regions are in contact with surrounding solvent, sequestering their hydrophobic tail regions towards the micelle center. The size and shape of a micelle depends upon the geometry of the constituent surfactant molecules and the solutions conditions, such as, surfactant concentration, temperature, PH, ionic strength and types of surfactant molecules in the solution [34]. Thus, the micelles of different sizes and shapes such as spherical, ellipsoidal, cylinders and bilayers could be formed and are in dynamic equilibrium depending upon the solutions conditions [34–36]. It is likely that micelles of different sizes and shapes, composed of Triton X-100 and Tween-20, formed a density gradient containing small size micelles towards the top and large size micelles towards the bottom of the gradient (Fig 3). A heterogenous protein sample containing a mixture of correctly folded, partially folded, incorrectly folded and aggregated protein molecules, dissolved in a buffer containing a heterogenous mixture of micelles, likely forms a heterogenous mix of protein-micelle complexes. It is likely that correctly folded protein molecules bind to small size micelles. In comparison, the misfolded and aggregated proteins expose hydrophobic surfaces and thus likely interact with large sizes micelles. Such a heterogenous protein-micelle complexes form a gradient containing the correctly folded and active protein molecules towards the top and aggregated proteins-micelle complexes towards the bottom of the gradient. This simple protein purification approach separates the active protein towards the top of the gradient, avoids aggregation and moderately stabilizes the protein, evident from the retention of biological activity despite numerous freeze thaw cycles (Fig 5). The yield of the purified protein by the old purification method and this new detergent gradient method is the same. We were able to purify about 4 mg of the protein by both the methods. However, 100% of the total purified protein by the old method became inactive by a single freeze thaw cycle. In comparison, about 50% of the total purified protein by the detergent gradient method retained activity and was stable despite several freeze thaw cycles. After further optimization, the assay will have significant impact upon the biotechnological industry and other fields of biological sciences.
